# A Modified Mucosal Incision for Easy Entry Into the Submucosal Space During Peroral Endoscopic Myotomy

**DOI:** 10.7759/cureus.72914

**Published:** 2024-11-03

**Authors:** Krishn K Rawal, Avval Sadikot, Chintan Kansagra, Chintan Mori

**Affiliations:** 1 Gastroenterology, Prime Institute of Digestive Sciences, Rajkot, IND; 2 Gastroenterology and Hepatology, Prime Institute of Digestive Sciences, Rajkot, IND; 3 Gastroenterology, Prime Institute of Digestive Sciences, Junagadh, IND

**Keywords:** achalasia cardia, dysphagia, endoscopic myotomy, esophageal motility disorders, poem

## Abstract

A technique of modified mucosal incision is discussed here for easy submucosal entry during peroral endoscopic myotomy (POEM). An L-shaped mucosal incision was used during peroral endoscopic myotomy in six cases of achalasia cardia. The incision was closed by clips in all the cases after the complete myotomy. Peroral endoscopic myotomy was successful in all six cases without any complication. The mucosal incision was closed by clips without any difficulty. The L-shaped mucosal incision for submucosal entry during peroral endoscopic myotomy made the procedure easy and quick.

## Introduction

Achalasia cardia is a motility disorder of the esophageal smooth muscle resulting in dysphagia due to the failure of the relaxation of the lower esophageal sphincter. There have been remarkable advances in the evaluation and treatment of achalasia cardia in the last decade. Peroral endoscopic myotomy (POEM) has revolutionized the management of achalasia cardia and other similar esophageal motility disorders. POEM is a type of natural orifice transluminal flexible endoscopic surgery where the myotomy of circular and longitudinal muscle fibers is done after creating a submucosal tunnel. It is now considered the first-line treatment modality for achalasia cardia, especially type III [1]. This concept of submucosal endoscopic esophageal myotomy was initially given by Pasricha et al. in 2007 [2]. Subsequently, Inoue et al. published the first case series of POEM in 17 consecutive patients with achalasia cardia [3]. The whole procedure of POEM comprises four basic steps: mucosal incision, submucosal tunnelling, myotomy, and the closure of the mucosal incision [4]. The most difficult and technically demanding step is the entry of an endoscope into the submucosal space after the mucosal incision. For beginners, often, it is disappointing and time-consuming. We hereby describe a technique of mucosal incision that would make the submucosal entry easier.

## Technical report

**Figure 1:**
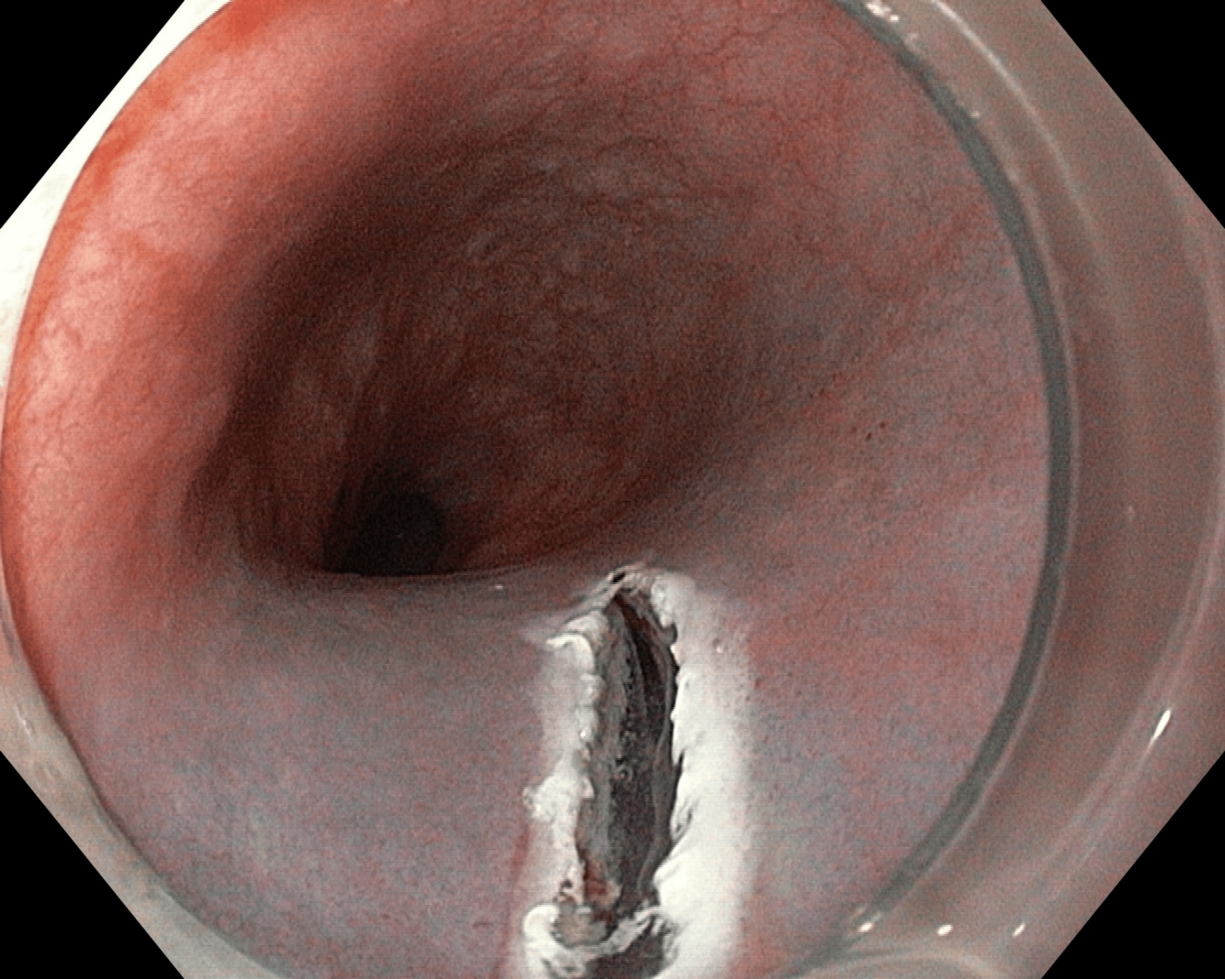
Conventional longitudinal mucosal incision for peroral endoscopic myotomy for entry into the submucosal space

**Figure 2:**
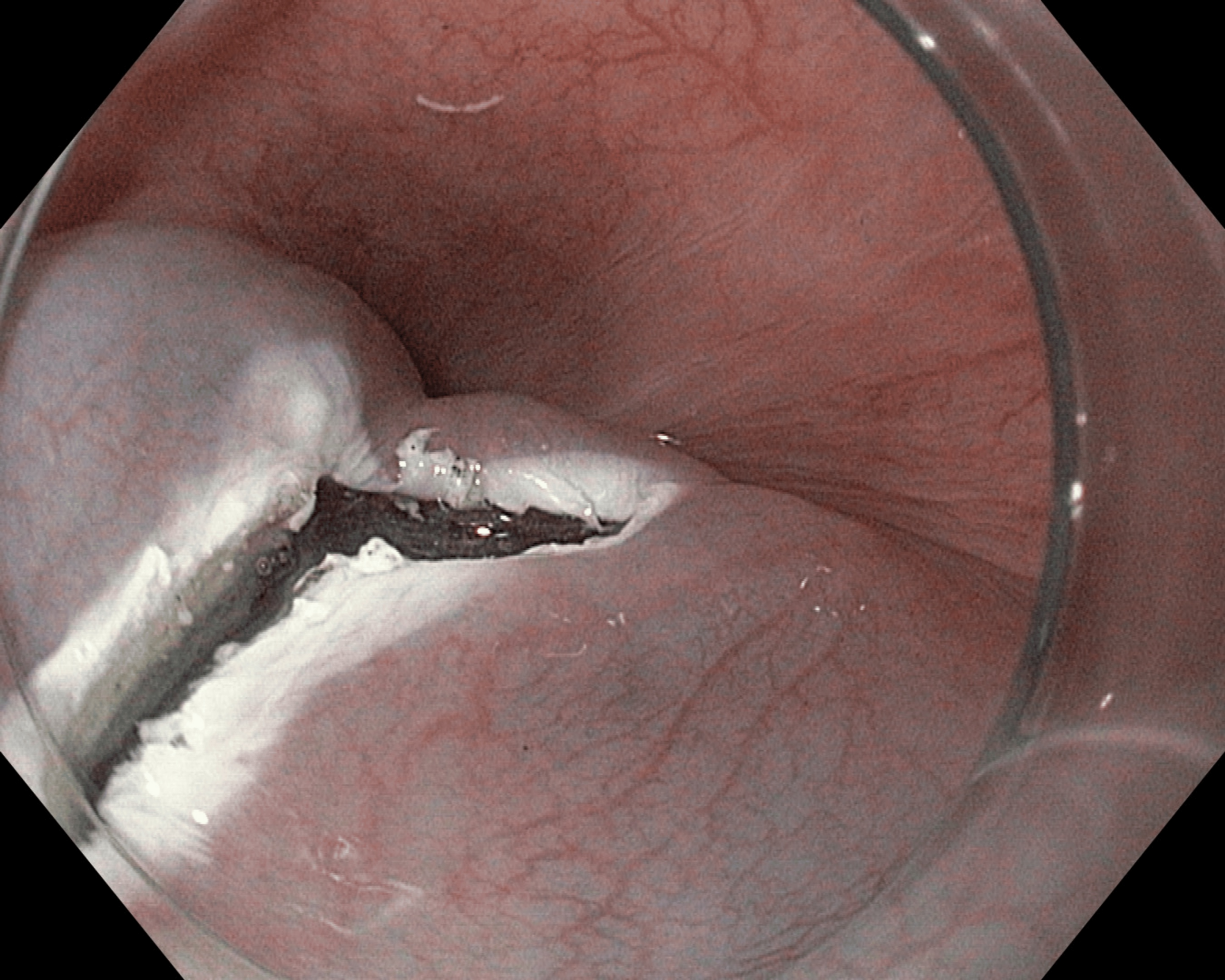
Longitudinal incision was extended for 3-4 mm transversely on anal end converting it to an L-shaped mucosal incision

A total of six patients with achalasia cardia were included in this pilot study. A written informed consent was obtained from all the patients. Achalasia cardia was diagnosed by upper gastrointestinal endoscopy, high-resolution esophageal manometry (HRM), and timed barium examination. All patients had presented with dysphagia. At baseline, the mean Eckardt score of all patients was 7.6, and the mean integrated relaxation pressure was 28 mmHg on HRM. None of the patients had a sigmoid esophagus. Endoscopy was done in all the patients 24 hours before POEM, and only clear liquids were allowed then after. Patients were kept nil by mouth overnight. Intravenous antibiotics were given on the day of the procedure and continued throughout the hospital stay. POEM was done in all the cases using this modified incision on mucosa. The instruments used were gastroscope (GIF-HQ190), triangle tip‐jet (TTJ) knife (KD-645L), injector (NM-600L 1YK 19), transparent distal attachment (D-201-11804), and coagrasper (FD-411QR), all from Olympus, Tokyo, Japan, and cautery (VIO 300 S) from ERBE, Tübingen, Germany. CO_2_ insufflation through a low-flow tube (MAJ-1742, Olympus, Tokyo, Japan) was used for the entire procedure. The mucosa was injected with a mixture of saline and indigo carmine to create a bleb approximately 8 cm above the gastroesophageal junction at two o'clock for anterior POEM and five o'clock for posterior POEM. Mucosa was incised in an L-shaped fashion in all the cases by a TTJ knife. Initially, a longitudinal incision of about 20 mm was made using Endocut Q (Figure 1). It was then extended transversely by 4-5 mm at the anal end on the right side, both in the anterior and posterior POEM (Figure 2). It was helpful to inject or flush with fluid at this point in one case (Video 1).

**Video 1:** After the injection of saline mixed with indigo carmine, a longitudinal incision was made on the mucosa The incision was then extended transversely for 3-4 mm to create an L-shaped mucosal incision. An endoscope entered the submucosal space without the need for undermining the edges of the incision. After submucosal dissection and myotomy, the mucosal incision was closed with clips

Submucosal dissection was done using spray coagulation. Vessels encountered during dissection were coagulated by coagrasper using soft coagulation. Submucosal tunnelling was continued for 2-3 cm beyond the lower esophageal sphincter. Myotomy was performed using spray coagulation beginning 2 cm below the incision and extending until the end of the tunnel. Lengths of esophageal and gastric myotomies performed were approximately 6 and 2 cm, respectively. The orientation of the myotomy was anterior in one case and posterior in five cases.

The closure of the mucosal incision was done by clips (EZ HX-610-090L, Olympus, Tokyo, Japan) in all the cases (Video 1). Out of six cases of achalasia cardia, five were type II, and one was type I based on manometric findings (Table 1).

**Table 1:** Demographic profiles, types of achalasia cardia, approach for myotomy, and complications noted during the procedure are compiled in this table

Case	Age (years)	Sex	Achalasia type	Myotomy approach	Complications
1	50	Male	II	Posterior	Nil
2	62	Male	I	Anterior	Nil
3	26	Male	II	Posterior	Nil
4	28	Female	II	Posterior	Nil
5	40	Male	II	Posterior	Nil
6	55	Female	II	Posterior	Nil

In all six cases, entry into the submucosal space using this technique was successful. The entry of an endoscope was possible in all the cases without undermining the edges by submucosal dissection at the apex of the incision at the anal end. An endoscope entered the submucosal space quickly with a median entry time of 1.15 minutes. The mucosal incision was closed in all cases by clips without any difficulty. In none of the cases, significant capnoperitoneum requiring needle aspiration was encountered. No complications such as mucosal injury, bleeding, and capnomediastinum were encountered in any of the cases. All six patients were admitted for observation and were given a liquid diet for 24 hours after confirming the absence of any leakage, clinically or radiologically. A proton pump inhibitor was given on discharge with a continuation of a soft diet for seven days.

## Discussion

**Figure 3:**
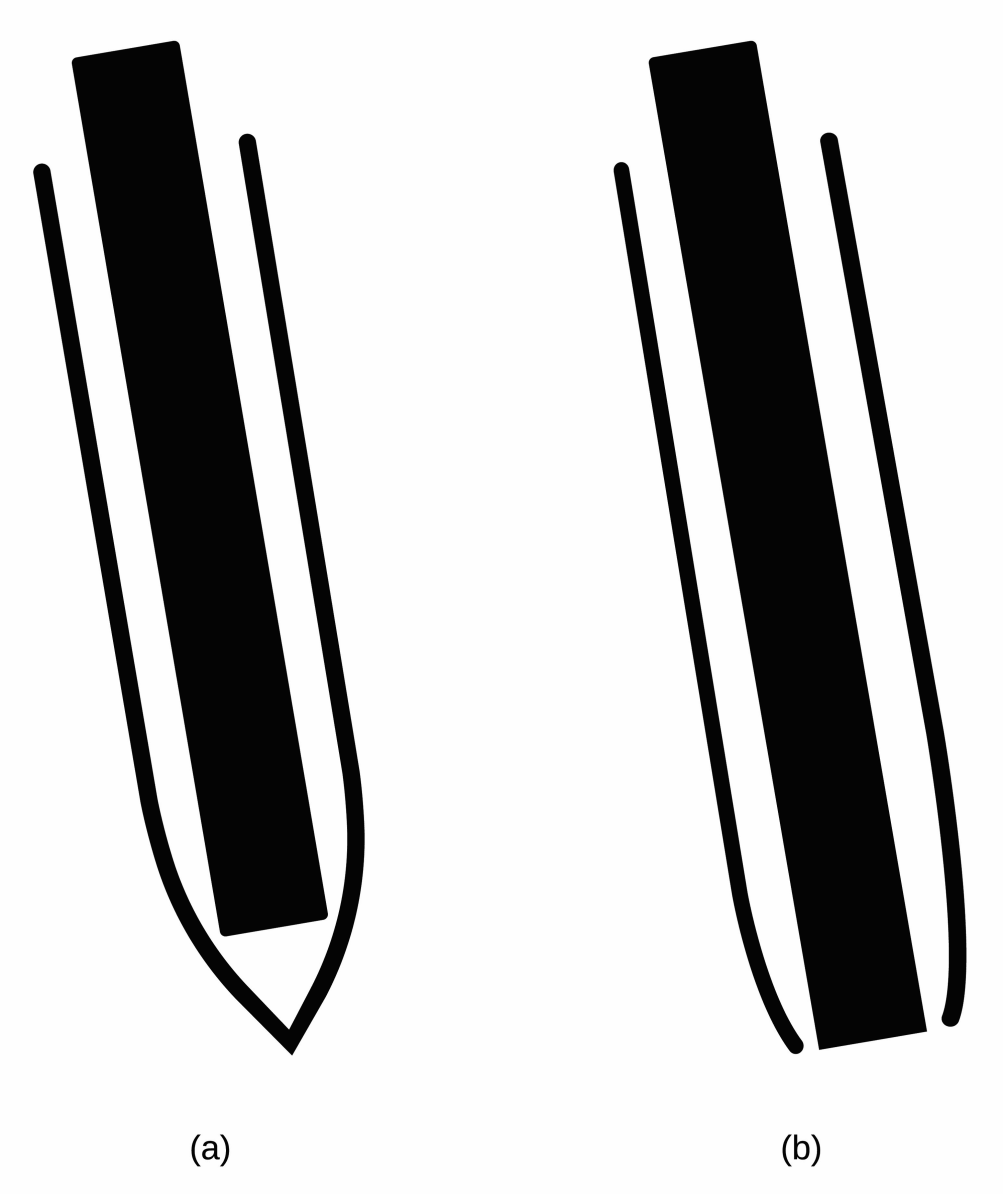
(a) Sharp angle at the apex of the mucosal incision increases the resistance to an endoscope for entry into the submucosal tunnel. (b) Blunt angle decreases the resistance to an endoscope for entry into the submucosal tunnel as it creates a wider space for the endoscope Image created and designed by Krishn Kant Rawal

The most difficult task in POEM is submucosal entry by an endoscope, particularly early in the learning phase. This is often frustrating and demanding. The factors that affect the difficulty of entering the submucosal tunnel include the operator's experience level, the length of the mucosal incision, the presence of submucosal fibrosis, and suboptimal lift during the procedure. Additionally, one of the modifiable factors is inadequate clearance of submucosal fibers along the margins of the incision. Traditionally, a longitudinal incision is made on the esophageal mucosa for entry into the submucosal space to create the tunnel. A longitudinal incision is easier to close but with the disadvantages of difficult entry into the tunnel and possibly increased risk of insufflation-related adverse events. This is because the endoscope is tightly encased by the incision. To overcome this, we propose a modified way of mucosal incision. The longitudinal arm (20 mm) of the mucosal incision was reinforced by a short transverse arm (4-5 mm). The sharp angle of this longitudinal incision was thus made blunt by a short transverse incision, and a wider space was created for endoscope entry into the submucosal space (Figure 3).

Even without undermining both the edges of the incision, the submucosal space was entered. Entry was quicker because time was not spent undermining the edges of the incision. Moreover, in the absence of the need for dissection at the entry point, the procedure became easier too. Flushing or injecting with fluid at this point could further help in opening the submucosal space. This also might prevent the accidental extension of longitudinal incision if mucosa was not healthy. In the case of thick mucosa with some submucosal fibrosis, this technique could be beneficial. The advantage of this new technique might be most significant during the operator's learning curve. Previously, transverse and inverted T-shaped incisions had been described from China resulting in easier entry and decreased insufflation-related adverse events [5,6]. A recent study also showed a trend toward a shorter time (median of two minutes) to enter into the submucosal space and a lesser incidence of gas-related adverse events with transverse incision, although none of these reached statistical significance [7]. However, any wider transverse extension of the incision would make the closure difficult with the clips. Therefore, these incisions could not become popular in general use. In our cases, since the transverse incision was very short, closure with clips was never an issue compared to the various types of incisions with regard to their pros and cons (Table 2).

**Table 2:** Comparison of various incisions

Type of incision	Ease of entry	Gas-related complications	Closure	Entry time
Longitudinal	Difficult	More	Easy	More
Transverse	Easy	Less	Difficult	Less
Inverted T	Easy	Less	Difficult	Less
L-shaped	Easy	Less	Easy	Less

The limitation of this pilot study could be the small number of cases. The small sample size (six patients) with the lack of randomized control limits the generalizability of the findings. Nevertheless, we presume this small modification (L-shaped incision) could make POEM a technically easier and quicker procedure, particularly for beginners. While the technique shows promise, its widespread implementation will depend on further validation from comparative studies involving larger cohorts from multiple centers.

## Conclusions

POEM is a technique that is constantly evolving. There are many confounders such as the type of mucosal incision, length of myotomy, orientation of myotomy, and preservation of sling fibers, which can be tailored. We propose a modification (L-shaped incision) to reduce the technical complexity and make the approach simple.
